# Supplementary material for: Itch/β-arrestin2-dependent non-proteolytic ubiquitylation of SuFu controls Hedgehog signalling and medulloblastoma tumorigenesis
Source: Nat Commun. 2018 Mar 7;9:976. doi: 10.1038/s41467-018-03339-0 (PMC5841288; doi:10.1038/s41467-018-03339-0)
Supplement: Supplementary file 1 — Supplementary Information [file 41467_2018_3339_MOESM1_ESM.pdf]

Manuscript Number: NCOMMS-16-11710B

Itch/βarrestin2-dependent non-proteolytic ubiquitylation of SuFu controls Hedgehog signalling and medulloblastoma tumourigenesis

Infante et al.

Supplementary Figures

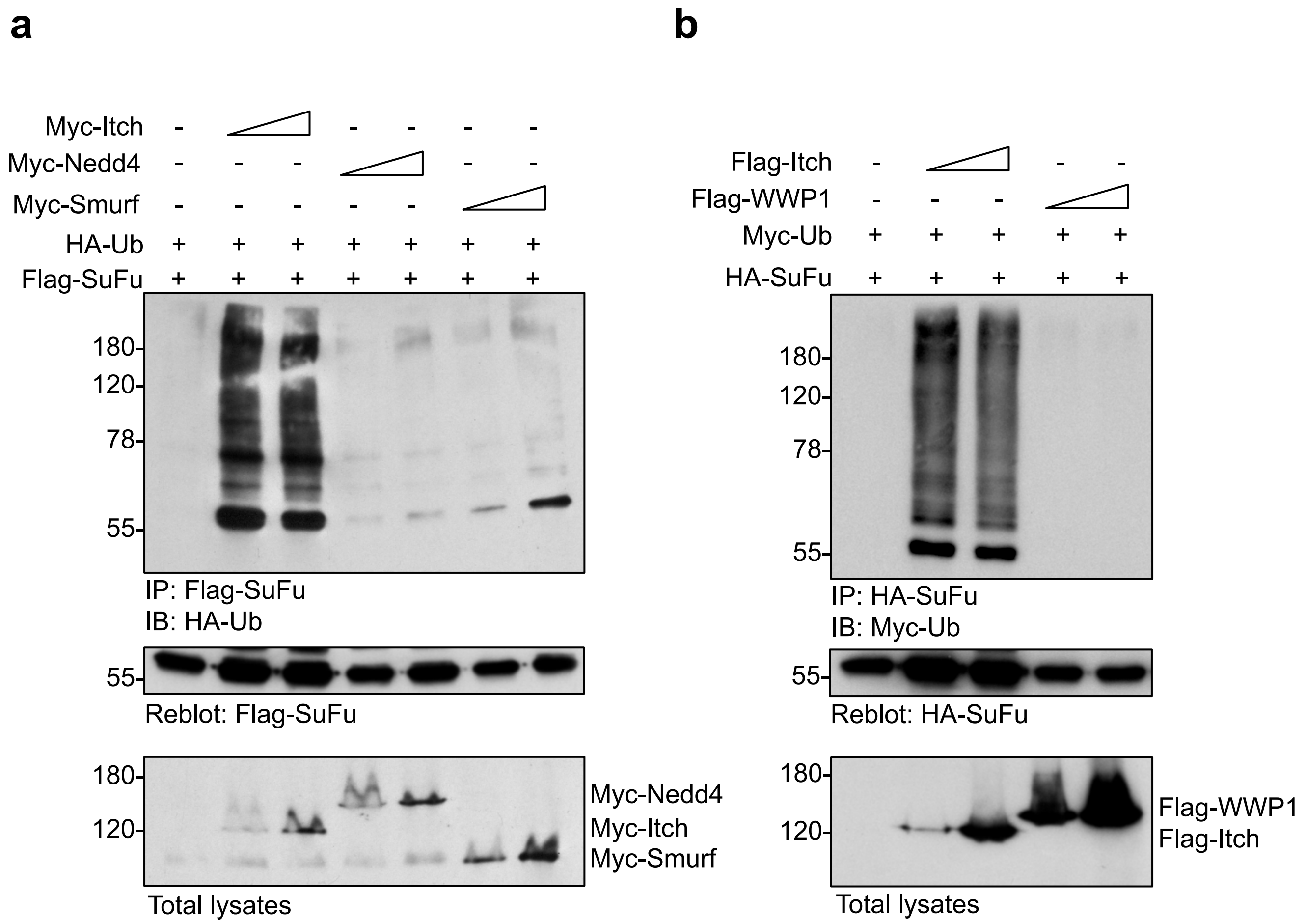

Supplementary Figure 1. SuFu is polyubiquitylated by Itch. (**a**,**b**) HEK293T cells were transfected with plasmids expressing HA-ubiquitin (HA-Ub) (**a**), or Myc-ubiquitin (Myc-Ub) (**b**) in presence of increasing amount of different HECT-type E3 ubiquitin ligases. Cell lysates were immunoprecipitated with an anti-Flag (**a**) or anti-HA antibody (**b**) and ubiquitylated forms were revealed with an anti-HA (**a**) or an anti-Myc antibody, respectively (**b**). The blot was reprobed with an anti-Flag (a) or an anti-HA antibody (b). Total protein levels are shown.

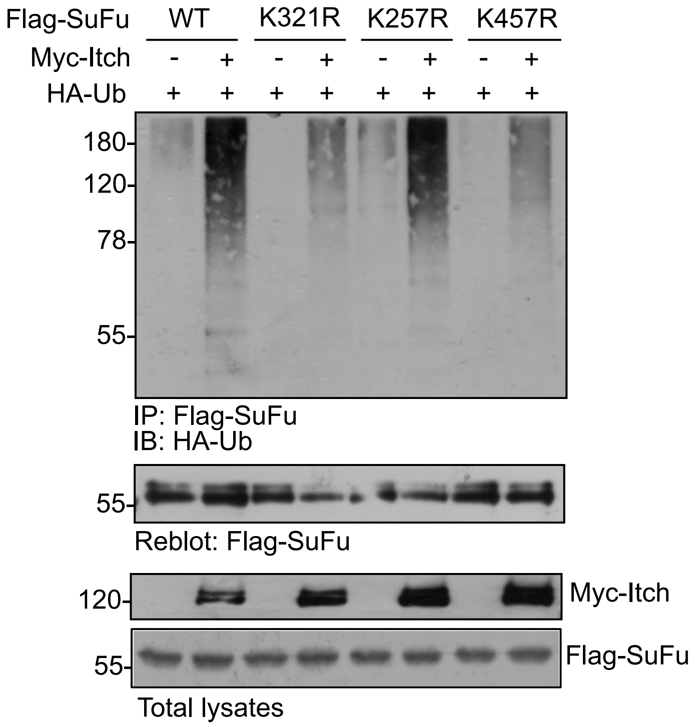

Supplementary Figure 2. **Itch-mediated SuFu ubiquitylation occurs on SuFu lysines 321 and 457.** Flag-SuFu WT or Flag-SuFu mutants (SuFu K321R, SuFu K257R or SuFu K457R) were co-transfected in HEK293T cells with HA-Ub in presence or absence of Myc-Itch. Cell lysates were immunoprecipitated with an anti-Flag antibody, followed by immunoblotting with an anti-HA antibody to detect conjugated HA-Ubiquitin. The blot was reprobed with an anti-Flag antibody. Total protein levels are shown.

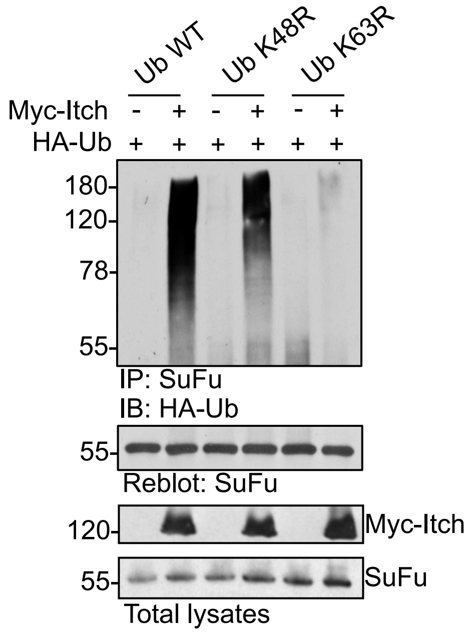

Supplementary Figure 3. **Itch-dependent SuFu ubiquitylation occurs through K63-mediated linkages.** HEK293T cells were co-transfected with HA-Ub WT or HA-Ub K48R or HA-Ub K63R mutants in presence or absence of Myc-Itch. Cell lysates were immunoprecipitated with an anti-SuFu antibody and ubiquitylated forms were revealed with anti-HA antibody. The blot was reprobed with an anti-SuFu antibody and total protein levels are shown.

	Free Energy of binding (kcal/mol) ± S.E.M.
SuFu WT	-46.11 ± 2.89
SuFu K321/457R	-45.41 ± 2.16

Table. Free energy of binding of Gli3 to SuFu wild-type and K321/457R double mutant, estimated by the MM-GBSA approach

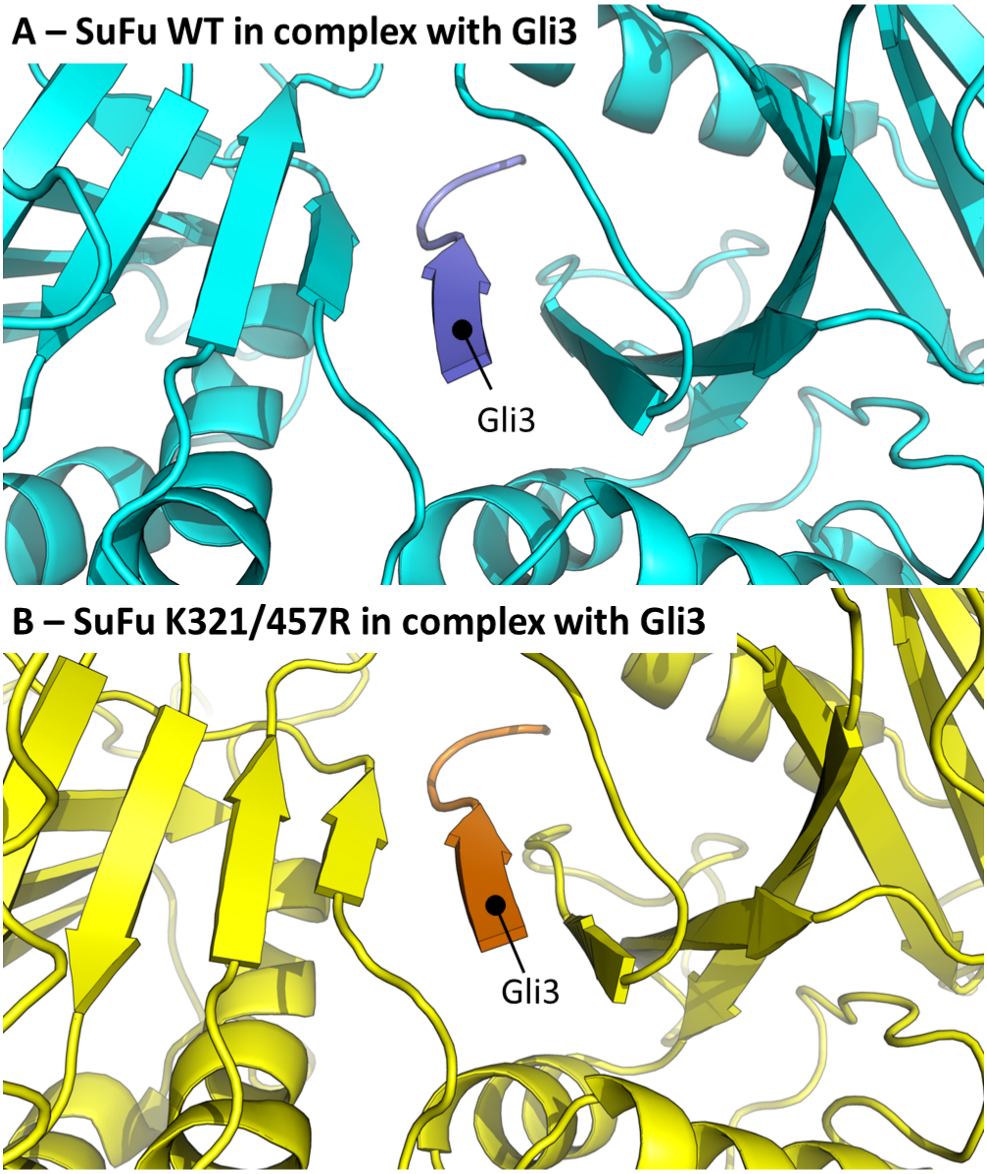

Supplementary Figure 4. Computational studies based on molecular dynamics (MD) simulations (200 ns of unrestrained trajectories) and calculations of the free energy of binding were performed by using the available crystallographic structure of SuFu in complex with a Gli3 peptide (PDB ID: 4BLD). Free energy of binding of Gli3 to SuFu WT and K321/457R mutant was estimated by the MM-GBSA approach, showing that both SuFu forms bind Gli3 with a highly comparable strength. Therefore, the K321/457R mutation does not alter the thermodynamic of Gli3 binding to SuFu. Conformational features of Gli3 binding site on SuFu WT and the SuFu K321/457R mutant were analyzed along MD trajectories. The most representative structure of SuFu WT-Gli3 and SuFu K321/457R-Gli3 complexes extracted from MD trajectories is shown in (**a**) and (**b**), respectively, suggesting that the overall geometry and architecture of the Gli3 binding site on SuFu is not altered by the K321/457R mutation.

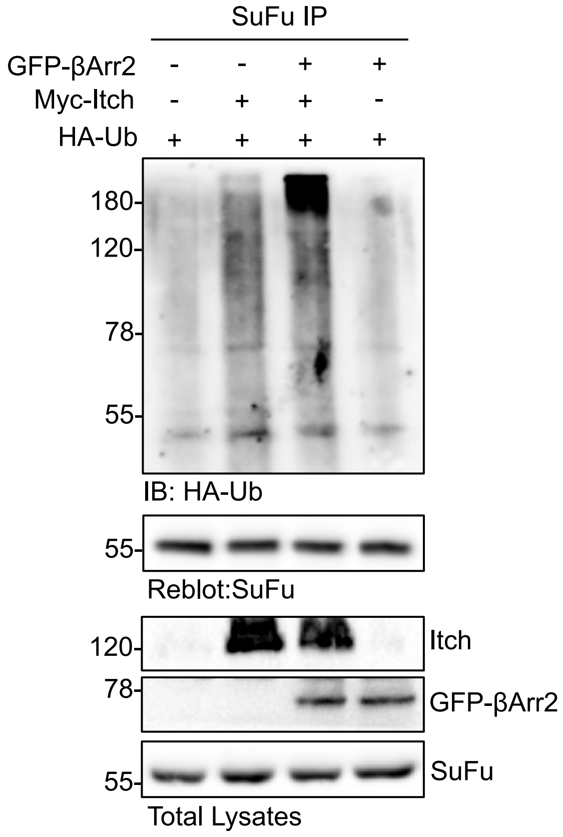

Supplementary Figure 5. β-arrestin2 increases the Itch-dependent ubiquitylation of SuFu. Itch$^{-/-}$ MEFs were transfected with HA-Ub in presence or absence of Myc-Itch or GFP-β-Arr2. Cell lysates were immunoprecipitated with an anti-SuFu antibody and ubiquitylated forms were revealed with anti-HA antibody. The blot was reprobed with an anti-SuFu antibody and total protein levels are shown.

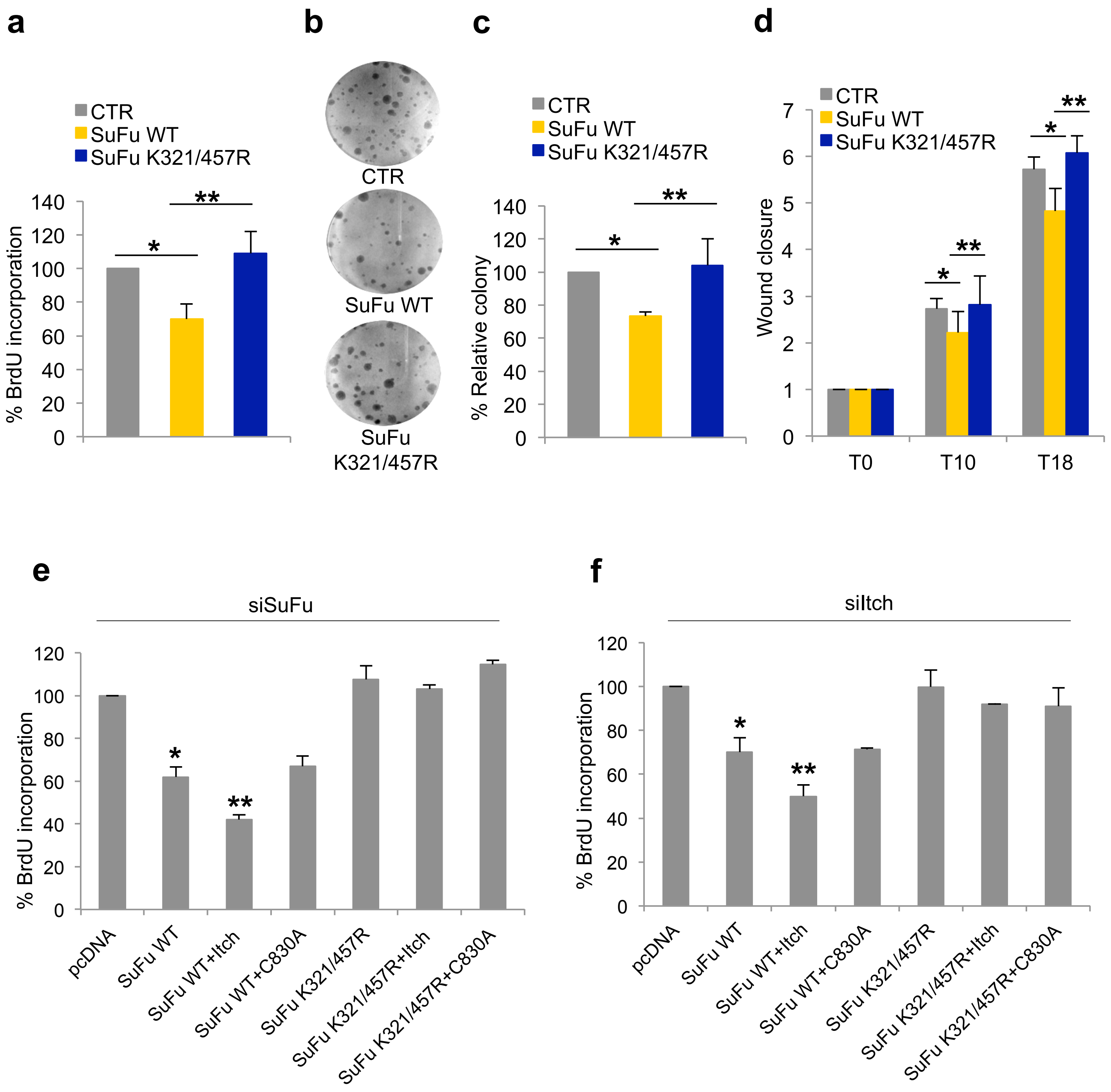

Supplementary Figure 6. (**a**) Effect of the SuFu K321/457R mutant on Daoy cell proliferation. The graph shows the percentage of bromodeoxyuridine (BrdU) incorporation in Daoy cells transduced with control lentivirus (CTR) or lentiviruses expressing SuFu WT or SuFu K321/457R. **P*< 0.05, SuFu WT versus CTR; ***P*< 0.05 SuFu K321/457R versus SuFu WT. (**b**,**c**) Colony formation assay in Daoy cells expressing SuFu WT or SuFu K321/457R or CTR. Representative images from experiments (**b**) and counts from the colony formation assay (**c**) are shown. **P*<0.05, SuFu WT versus CTR; ***P*<0.05, SuFu K321/457R versus SuFu WT. (**d**) Wound healing assay in Daoy cells expressing SuFu WT or SuFu K321/457R or CTR. The graph represents the relative wound closure at 0 (T0), 10 (T10), 18 (T18) hours. **P*<0.05, SuFu WT versus CTR; ***P*<0.05 SuFu K321/457R versus SuFu WT. (**e**,**f**) BrdU assays in Daoy cells transfected with SuFu siRNA (siSuFu) (**e**) or Itch siRNA (siItch) (**f**) in combination with SuFu WT or SuFu K321/457R in presence or absence of Itch WT (Itch) or ItchC830A mutant (C830A). **P*<0.05, SuFu WT versus pcDNA and ***P*<0.01, SuFu WT+Itch versus SuFu WT in (**e**). **P*<0.05, SuFu WT versus pcDNA and ***P*< 0.05, SuFu WT+Itch versus SuFu WT in (**f**). Error bars indicate SD from at least three independent experiments. *P* values were determined using Student's *t*-test.

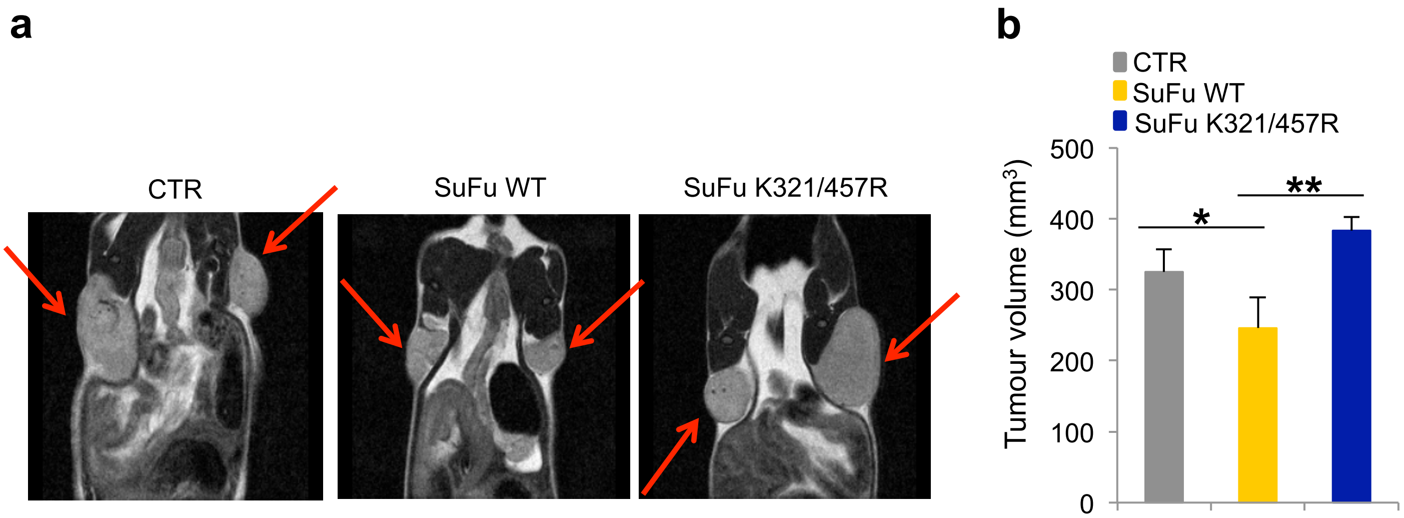

Supplementary Figure 7. (**a**) MRI imaging. Axial tumour imaging analysis performed using a 1T MRI scanner (Bruker, Icon, Germany) 41 days after implantation of a human Daoy MB cell-derived tumour in NOD/SCID mice. (**b**) Tumour volume evaluation by MRI analysis. Tumour volume was measured 41 days after implantation. **P*<0.01, SuFu WT versus CTR; ***P* <0.01, SuFu K321/457R versus SuFu WT. Error bars indicate SD. *P* values were determined using Mann Whitney *U*-test.

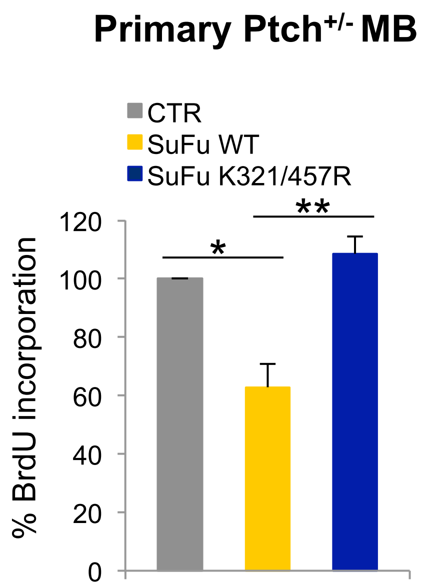

Supplementary Figure 8. Effect of the SuFu K321/457R mutant on primary Ptch$^{+/-}$ MB cells. The graph shows the percentage of BrdU incorporation in primary medulloblastoma cells from Ptch$^{+/-}$ mice transduced with control lentivirus (CTR) or lentiviruses expressing SuFu WT or SuFu K321/457R. Error bars indicate SD from three independent experiments. *P<0.05, SuFu WT versus CTR and **P<0.05, SuFu K321/457R versus WT (Student's *t*-test).

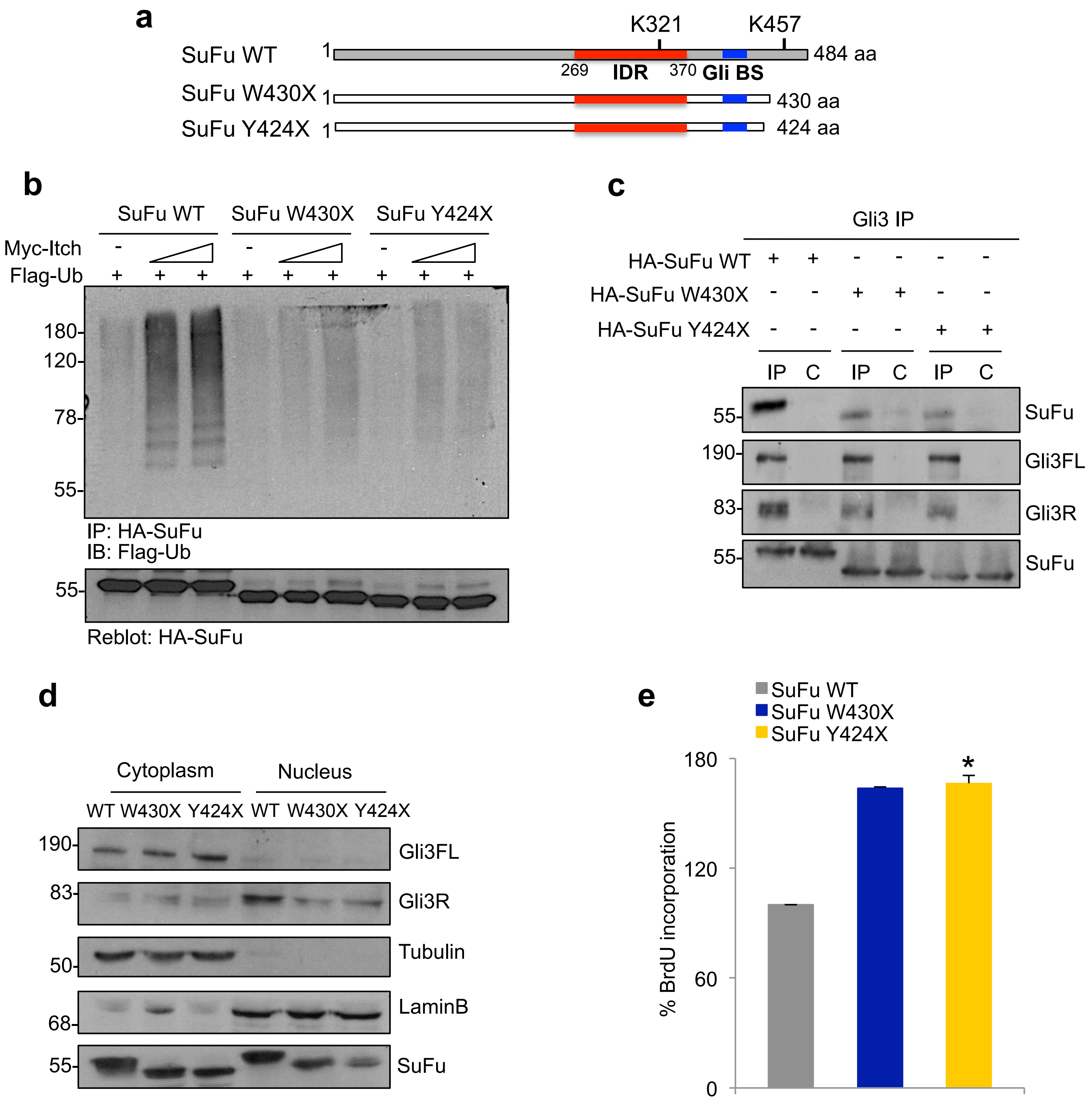

Supplementary Figure 9. Itch-dependent SuFu ubiquitylation is impaired in SuFu infant Shh-MBs mutants. (**a**) Schematic representation of SuFu WT and SuFu Y424X and SuFu W430X mutants. (**b**) HEK293T cells were co-transfected with Flag-Ub and HA-SuFu WT or HA-SuFu W430X or HA-SuFu Y424X mutants and with increasing amount of Myc-Itch. Cell lysates were immunoprecipitated with anti-HA and ubiquitylated forms were revealed with anti-Flag antibody. The blot was reprobed with anti-HA antibody. (**c**) Association between endogenous Gli3 and HA-SuFu WT or HA-SuFu W430X or HA-SuFu Y424X mutants. Cell lysates from MEF cells were immunoprecipitated with anti-Gli3 antibody or control goat antisera (IgG) and immunoblotted with anti-HA antibodies. The blot was reprobed with anti-Gli3 antibody. Total protein levels are shown. (**d**) Subcellular fractions generated from MEF cells transfected with HA-SuFu WT or HA-SuFu W430X or HA-SuFu Y424X plasmids. Gli3FL, Gli3R and SuFu protein levels were determined separately in cytoplasmic and nuclear fractions. Lamin B and Tubulin were respectively used as nuclear and cytoplasmic control to assess the quality of the fractionation. (**e**) Effect of SuFu Y424X and SuFu W430X mutants on Daoy cell proliferation. The graph shows the percentage of BrdU incorporation in Daoy cells transfected with indicated plasmids. Error bars indicate SD from three independent experiments. *$P<0.05$, SuFu Y424X and SuFu W430X versus SuFu WT (Student's *t*-test).

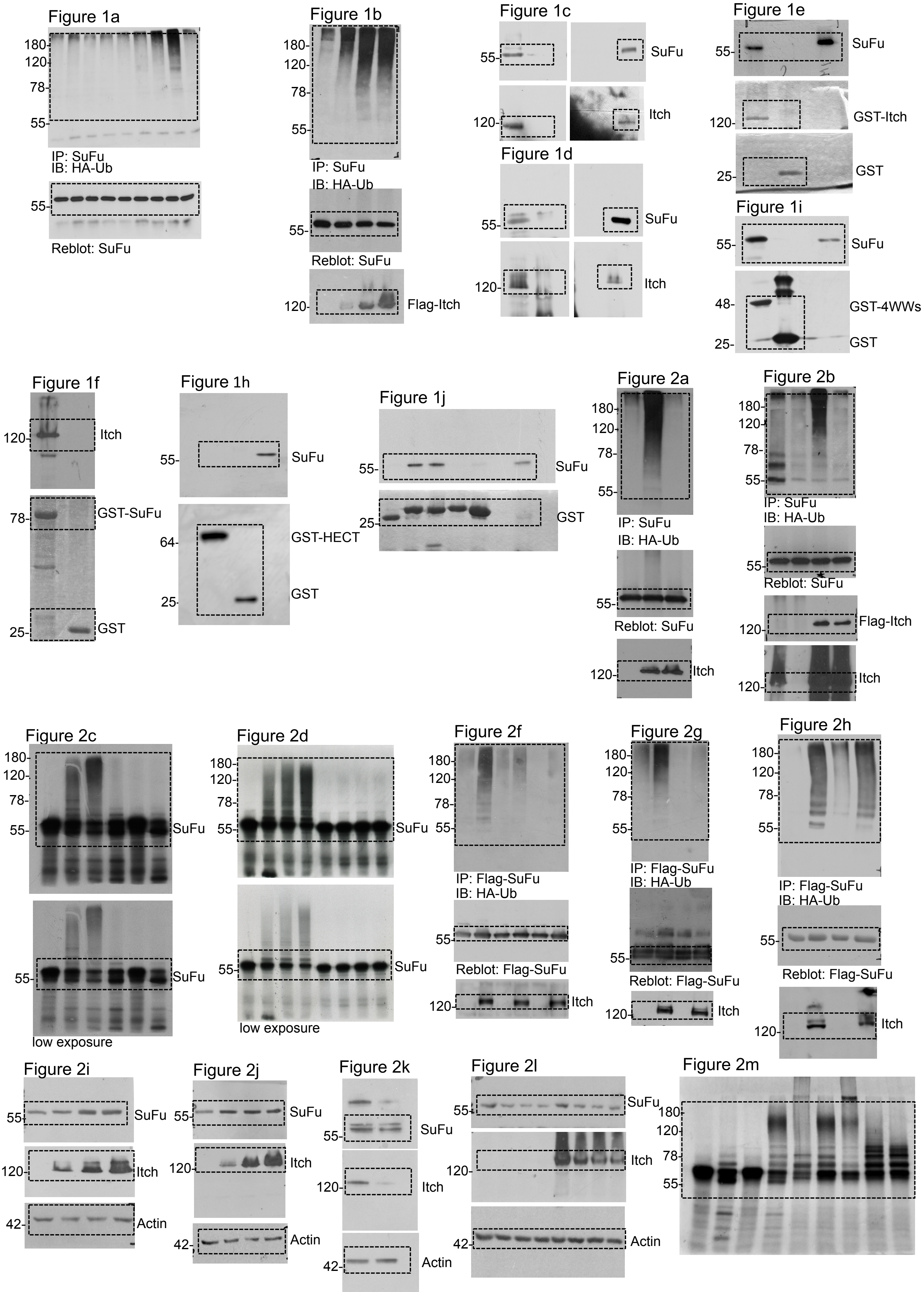

Supplementary Figure 10. Uncropped blots and gels data for Figures

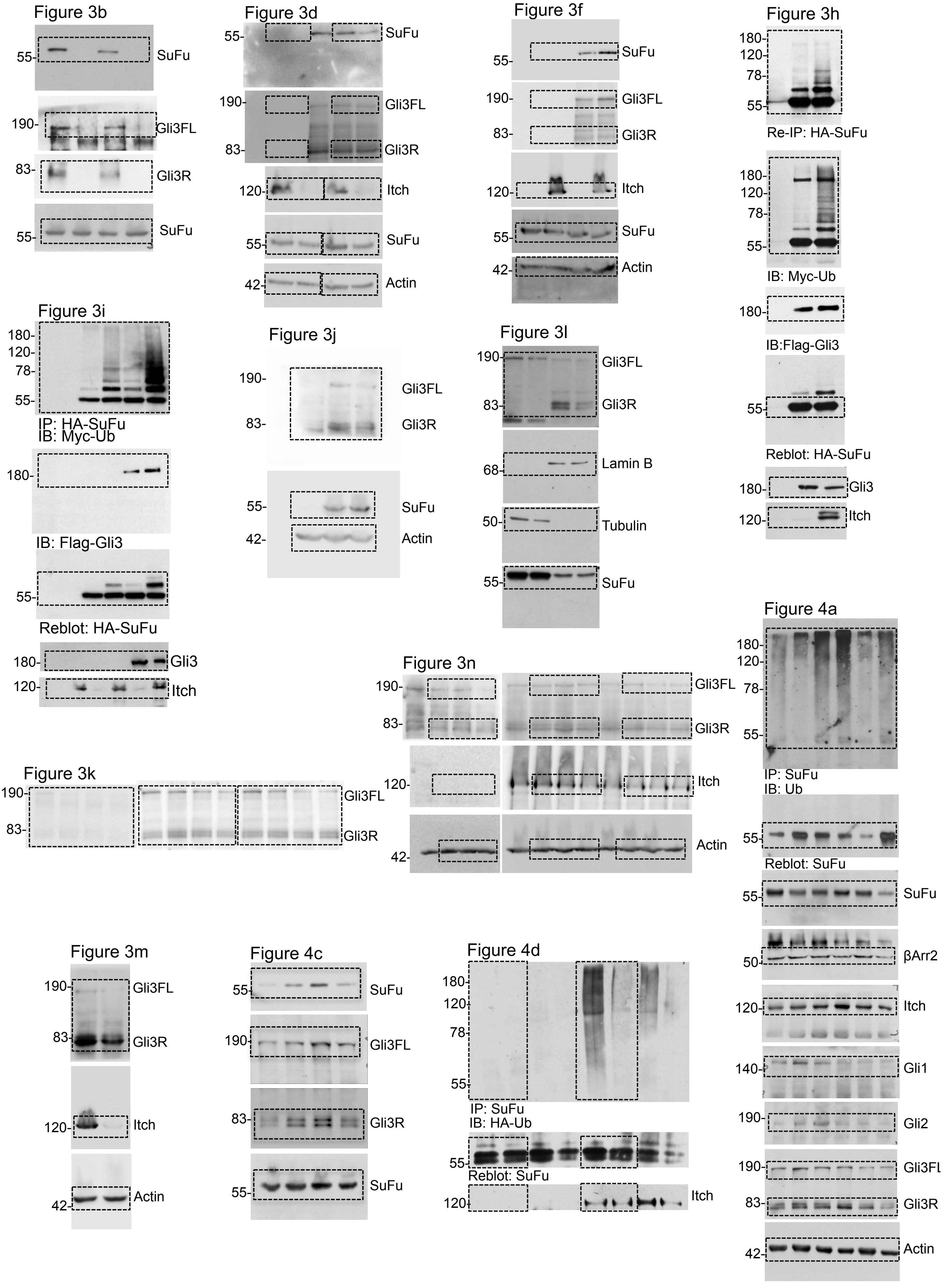

Supplementary Figure 11. Uncropped blots and gels data for Figures

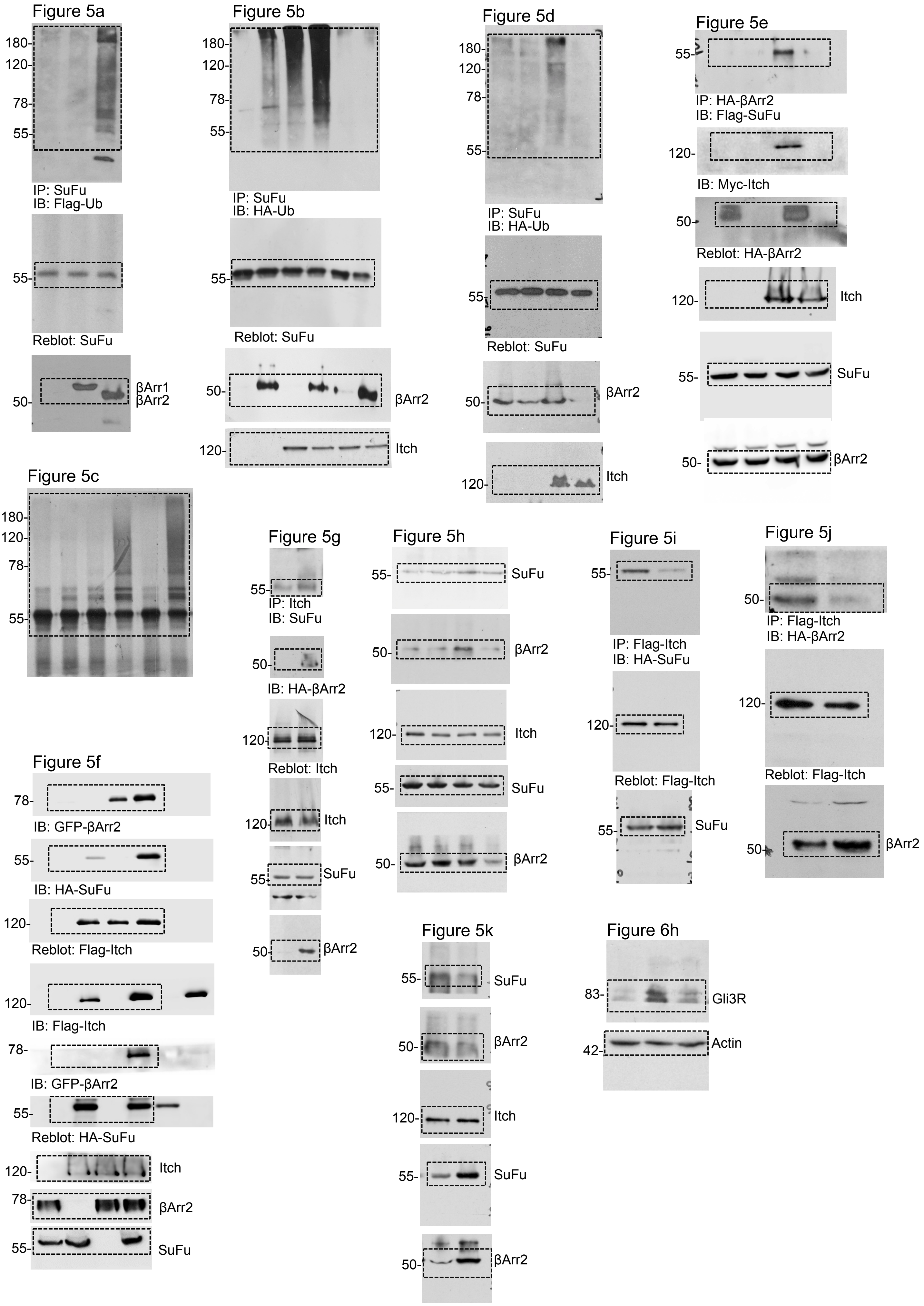

Supplementary Figure 12. Uncropped blots and gels data for Figures

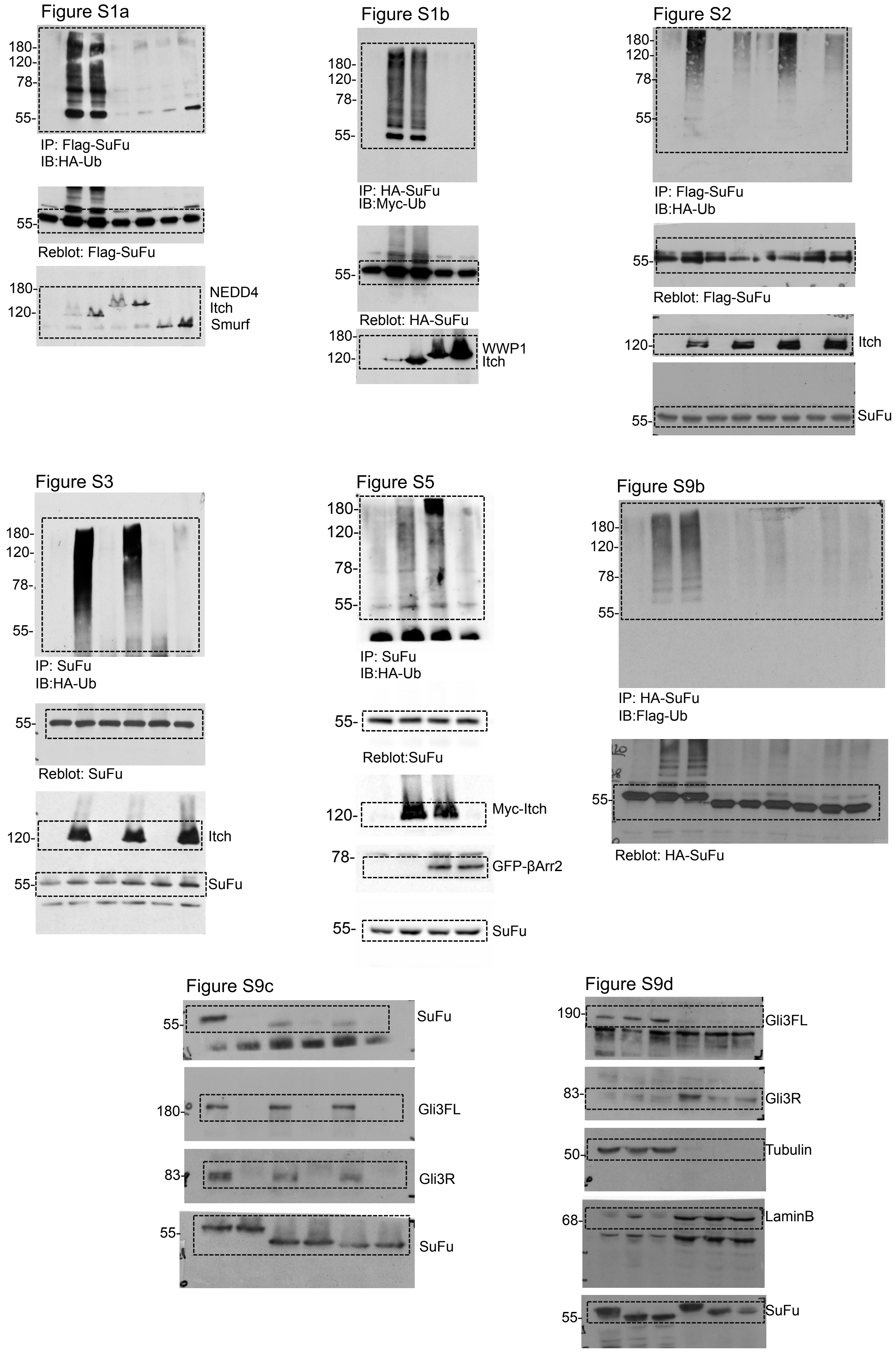

Supplementary Figure 13. Uncropped blots and gels data for Figures
